# Gamma-Camera Direct Imaging of the Plasma and On/Intra Cellular Distribution of the ^99m^Tc-DPD-Fe_3_O_4_ Dual-Modality Contrast Agent in Peripheral Human Blood

**DOI:** 10.3390/ma17020335

**Published:** 2024-01-09

**Authors:** Maria-Argyro Karageorgou, Adamantia Apostolopoulou, Mina-Ermioni Tomazinaki, Dragana Stanković, Efstathios Stiliaris, Penelope Bouziotis, Dimosthenis Stamopoulos

**Affiliations:** 1 Department of Physics, School of Science, National and Kapodistrian University of Athens, 15784 Athens, Greece; ermina@phys.uoa.gr (M.-E.T.); stiliaris@phys.uoa.gr (E.S.); 2 Institute of Nuclear & Radiological Sciences & Technology, Energy & Safety, National Center for Scientific Research “Demokritos”, 15341 Athens, Greece; a.apostolopoulou@rrp.demokritos.gr (A.A.); bouzioti@rrp.demokritos.gr (P.B.); 3 Laboratory of Biology, School of Medicine, Department of Basic Medical Sciences, National and Kapodistrian University of Athens, 11527 Athens, Greece; 4 Laboratory for Radioisotopes, “Vinča” Institute of Nuclear Sciences, University of Belgrade, P.O. Box 522, 11001 Belgrade, Serbia; dragana.s@vin.bg.ac.rs

**Keywords:** iron oxide nanoparticles, technetium-99m, dual-modality contrast agent, gamma-camera imaging, peripheral human blood, cellular distribution, biocompatibility

## Abstract

The radiolabeled iron oxide nanoparticles constitute an attractive choice to be used as dual-modality contrast agents (DMCAs) in nuclear medical diagnosis, due to their ability to combine the benefits of two imaging modalities, for instance single photon emission computed tomography (SPECT) with magnetic resonance imaging (MRI). Before the use of any DMCA, the investigation of its plasma extra- and on/intra cellular distribution in peripheral human blood is of paramount importance. Here, we focus on the in vitro investigation of the distribution of ^99m^Tc-DPD-Fe_3_O_4_ DMCA in donated peripheral human blood (the ligand 2-3-dicarboxypropane-1-1-diphosphonic-acid is denoted as DPD). Initially, we described the experimental methods we performed for the radiosynthesis of the ^99m^Tc-DPD-Fe_3_O_4_, the preparation of whole blood and blood plasma samples, and their incubation conditions with ^99m^Tc-DPD-Fe_3_O_4_. More importantly, we employed a gamma-camera apparatus for the direct imaging of the ^99m^Tc-DPD-Fe_3_O_4_-loaded whole blood and blood plasma samples when subjected to specialized centrifugation protocols. The direct comparison of the gamma-camera data obtained at the exact same samples before and after their centrifugation enabled us to clearly identify the distribution of the ^99m^Tc-DPD-Fe_3_O_4_ in the two components, plasma and cells, of peripheral human blood.

## 1. Introduction

In recent decades, radiolabeled iron oxide nanoparticles have attracted tremendous interest in the diagnosis of diseases due to their dual-imaging potential. Concretely, they can act as dual-modality contrast agents (DMCAs) for single photon emission computed tomography (SPECT) (or positron emission tomography (PET)) and magnetic resonance imaging (MRI) applications, since they are able to combine the benefits of each of the aforementioned imaging techniques, leading to an accurate and improved diagnostic result, which is superior to the one obtained from each imaging technique separately. For example, among the advantages of these techniques are the high sensitivity of nuclear imaging, namely SPECT (or PET) and the high spatial resolution of MRI [1,2,3,4]. To this effect, many studies [5,6,7,8,9,10,11] have highlighted the enhanced diagnostic efficacy of SPECT (or PET)/MRI DMCAs compared to conventional contrast agents. In everyday clinical practice, radionuclides are used extensively up to now in the form of radiopharmaceuticals both for diagnostic and for therapeutic purposes. For this reason, the departments of Nuclear Medicine in hospitals are fully equipped with all the appropriate special facilities and infrastructure for daily use of radioactive agents. Of course, the use of other alternative choices, such as fluorescent probe-based DMCAs, in biomedical applications constitutes an interesting suggestion for research and for this reason, many studies refer to such probes in the literature [12,13,14]. 

Following intravenous administration, DMCAs enter the bloodstream to reach target tissues, coming into direct contact with all blood cellular components—red blood cells (RBCs), white blood cells (WBCs), and platelets (PLTs). According to many studies, the DMCAs’ physicochemical characteristics, which mainly include their size and surface functionalization, affect the ability of the DMCA to be internalized by cells through mechanisms like endocytosis or diffusion in a passive manner [15,16,17,18,19]. While iron oxide-based DMCAs are generally considered biocompatible [20,21,22,23,24], upon entering blood cells, they may induce toxicity primarily through the production of reactive oxygen species (ROS). Such effects are detrimental, since they can cause damage in the erythrocytes’ membrane, abnormalities in the cells’ shape (i.e., the cells may appear without their normal biconcave shape, but they may show abnormal forms such as echinocytes or acanthocytes) or hemolysis [25,26,27,28,29,30,31,32]. Accordingly, the preservation of both the biological structure and function of all blood cellular constituents upon interaction with a DMCA is crucial for its potential in vivo application in diagnosis.

Furthermore, the attachment or internalization of a DMCA in the blood cells, will result in its non-specific distribution in various organs of the human body, a fact that will be detrimental for its use as a selective contrast agent in diagnostic imaging. At this point, we should stress many in vitro experimental procedures have been performed to determine the intracellular and extracellular distribution of nanoparticles, including inductively coupled plasma mass spectrometry (ICPMS) [33], bioluminescence reaction [34], chemical etching [35], etc. 

To this effect, our aim is to investigate the distribution of a particular DMCA in the two components, plasma and cells, of peripheral human blood donated from five healthy donors. The DMCA we refer to consists of Fe_3_O_4_ nanoparticles, which are surface-functionalized with the ligand 2-3-dicarboxypropane-1-1-diphosphonic-acid (DPD) and radiolabeled with technetium-99m (^99m^Tc), resulting in ^99m^Tc-DPD-Fe_3_O_4_. For this goal, the DMCA was incubated at room temperature for 2 h with: (i) whole blood which underwent a two-stage purification with DMCA-free poor platelets plasma (PPP), (ii) PPP per se and (iii) intact whole blood. Then, we employed a gamma-camera apparatus for the direct imaging of the samples when subjected to specialized centrifugation. Specifically, all samples were imaged before and after centrifugation at 1200× *g* for 10 min. The direct comparison of the gamma-camera data obtained from the exact same samples before and after their centrifugation enabled us to identify the distribution of the ^99m^Tc-DPD-Fe_3_O_4_ in the two components, plasma and cells, of the parent samples of peripheral human blood. Our data show clear evidence the DMCA stays dissolved in the human plasma and does not attach/enter the cellular components of peripheral human blood. 

## 2. Materials and Methods

### 2.1. Materials

As a radionuclide emitting gamma radiation, ^99m^Tc poses serious health threats. Consequently, all the ^99m^Tc-based radiochemical processes were performed under relevant radioprotection to minimize the possibility of harm and ensure the safety of the involved scientists. To this effect, our study was carried out in authorized radiochemical laboratories, each equipped with the necessary infrastructure, licenses, and expertise to safely perform experiments with radionuclides.

All reagents and solvents used for radiolabeling of DPD-Fe_3_O_4_ with ^99m^Tc and the appropriate equipment employed to determine its radiolabeling yield are described in detail in the respective section of our previous study, which can be found in [36]. 

### 2.2. Radiolabeling of Fe_3_O_4_-DPD with ^99m^Tc Radionuclide

The synthesis and basic characterization (including crystallographic, morphologic, magnetic, etc.) of the parent contrast agent (CA), DPD-Fe_3_O_4_, have been reported in a previous study of ours [37]. 

Exact details on the radiolabeling process of DPD-Fe_3_O_4_ with ^99m^Tc, along with the calculation of the radiolabeling yield of the obtained DMCA can be found in [36]. At this point, we should note for this study 140 μL of DPD-Fe_3_O_4_ (obtained from a sample of initial concentration C_CA_ = 6 mg/mL) were radiolabeled with 100 μL of Na^99m^TcO_4_^−^ (of average activity ~2.5 mCi/100 μL). Furthermore, data on the hydrodynamic size of the DMCA, ^99m^Tc-DPD-Fe_3_O_4_, along with in vitro radiolabeling stability data on different biological media can also be found in [36]. 

### 2.3. Healthy Donors and Peripheral Whole Blood Collection

Five donors participated in this study (see the respective information for all donors in Table 1). Before the beginning of the experimental process, the donors provided a complete blood count examination (see the respective hematological data of all five donors in Table 2, below) to investigate their health record and hence their eligibility for participation. None of the five donors showed any record of hematological or current/chronic diseases; they were regarded as healthy.

Following the Ethics Committee of the National Centre for Scientific Research “Demokritos” approval (EHDE 17/21-02-2023), the five donors underwent blood sampling. All the experiments in our study were performed according to relevant guidelines and regulations. 

For the realization of our study, 9 mL of peripheral whole blood were obtained by venipuncture from each donor and were equally distributed in three anticoagulant-containing ethylenediaminetetraacetic acid (EDTA) K3 test tubes. All blood samples were used immediately after their collection [24].

### 2.4. Preparation of Samples

Ideally, when hosted in a biological sample, for instance, human whole blood, the DMCA, ^99m^Tc-DPD-Fe_3_O_4_, should be colloidally stable exhibiting negligible precipitation even during centrifugation of the biological sample. On the other hand, during the preparation of the DMCA, that is at the stage where the DPD-Fe_3_O_4_ is radiolabelled, centrifugation may be used as an efficient means to purposely precipitate the extra DMCA amount of an oversaturated solution so that a colloidally stable sample is finally achieved. For these reasons, at the first stage of our experiments, we investigated the centrifugation conditions under which the DMCA precipitated. Accordingly, a reference DMCA solution was centrifuged for 10 min at the maximum value, 3350× *g*, that we could use in our laboratory (we noted the cellular components of whole blood precipitate completely at a much lower value, 1200× *g*). In some cases after centrifugation at 3350× *g*, the DMCA was mildly fractionated, forming a small pellet at the bottom of the Eppendorf. The radioactivities of both the supernatant and the pellet were measured by means of a Capintec dose calibrator and compared to the initial radioactivity of the DMCA (measured before centrifugation). Since the radioactivity of the pellet was lower than 4% of the initial radioactivity of the DMCA, we considered the supernatant hosted a practically soluble DMCA. Such purified and colloidally stable DMCA supernatant samples are used for the incubation with peripheral whole blood following the procedure discussed below. 

Initially, 1800 μL of whole blood was isolated from the EDTA test tubes and was equally distributed in two Eppendorfs. Then, the EDTA test tubes were centrifuged at 1200× *g* for 10 min so that poor-platelets plasma (PPP) could be isolated. Thus, after centrifugation, 900 μL PPP was collected and also placed in an Eppendorf. Then, each one of the three Eppendorfs was mixed with 100 μL of ^99m^Tc-DPD-Fe_3_O_4_ DMCA and was stirred gently for 120 min at room temperature. Before proceeding, we termed the Eppendorfs as: Eppendorf (1) the one containing intact whole blood incubated with the DMCA, Eppendorf (2) the one containing PPP incubated with the DMCA, and Eppendorf (3) the one containing intact whole blood incubated with the DMCA. 

After the incubation of the samples, the Eppendorf (1) was subjected to an additional processing step: a two-stage purification by centrifugation (1200× *g* for 10 min), removal of its supernatant PPP, and replacement with an equal amount of pristine autologous PPP. Thus, the following samples were finally prepared: whole blood that was originally incubated with the DMCA and finally twice-purified with pristine PPP (Eppendorf (1)), PPP incubated with the DMCA (Eppendorf (2)) and intact whole blood incubated with the DMCA (Eppendorf (3)). Identical control samples were prepared following the above processes by using ^99m^Tc instead of DMCA.

### 2.5. Gamma-Camera Imaging of Samples

Following the preparation of the samples discussed above, gamma-camera imaging was conducted by employing a small-field gamma-camera system, which was used for animal and other preclinical studies. Its spatial resolution is 0.95 ± 0.05 mm on planar imaging. Exact details on the gamma-camera system can be found in [38,39]. 

For the conduct of the imaging study, each one of the samples of the Eppendorfs (1), (2), and (3) was loaded on a syringe (1 mL insulin syringe with 27G x ½” inch mounted needle, Safety A.T/G.), also termed (1), (2), and (3), which served as the sample holder. Thus, the following syringes were finally prepared and imaged: whole blood that was originally incubated with the DMCA and finally twice-purified with pristine autologous PPP (syringe (1)), PPP incubated with the DMCA (syringe (2)), and intact whole blood incubated with the DMCA (syringe (3)). 

**Figure 1:**
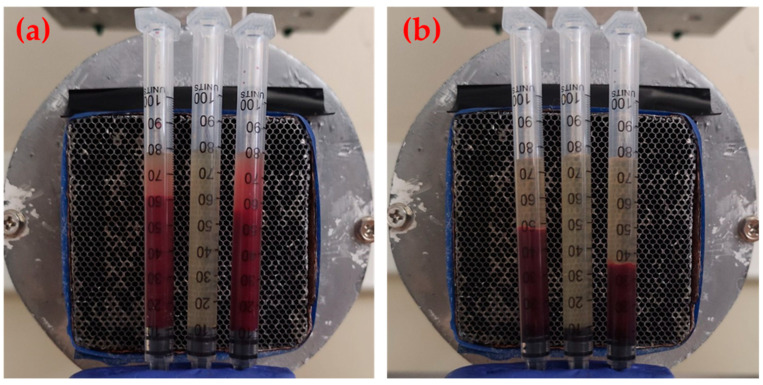
Gamma-camera imaging of twice-purified whole blood originally incubated with DMCA (syringe (1)-left), PPP incubated with the DMCA (syringe (2)-middle), and intact whole blood incubated with the DMCA (syringe (3)-right), (**a**) before and (**b**) after centrifugation. In panel (**a**) it is evident that mild precipitation of cells under the gravity force during imaging is inevitable.

Each syringe was loaded by carefully adding the respective sample with a micropipette until it was approximately filled up to 0.8 mL. In this way, its content was adjusted to the gamma-camera vertical field of view (48 mm) (Figure 1a). Then, each set of three syringes (1), (2), and (3) were fixed in an upright position and placed for imaging (Figure 1a). At the end of the first imaging, the syringes were centrifuged at 1200× *g* for 10 min and immediately placed for a second imaging (Figure 1b). In the rest of the paper the data of the first imaging are termed “before centrifugation”, while those of the second imaging are termed “after centrifugation”.

### 2.6. Statistical Analysis

Although the sample of donors was small (five donors), consistent results were obtained from all donors, as indicated by this introductory study. The obtained results were analyzed statistically using a correlation test. 

## 3. Results and Discussion

Before experimentation, the donors provided a complete blood count to examine their health record and investigate their eligibility for participation. The hematological data of all donors are provided in Table 2, above. According to these results, all hematological parameters were within the physiological range, thus the donors were considered to be healthy and were eligible for participation.

**Figure 2:**
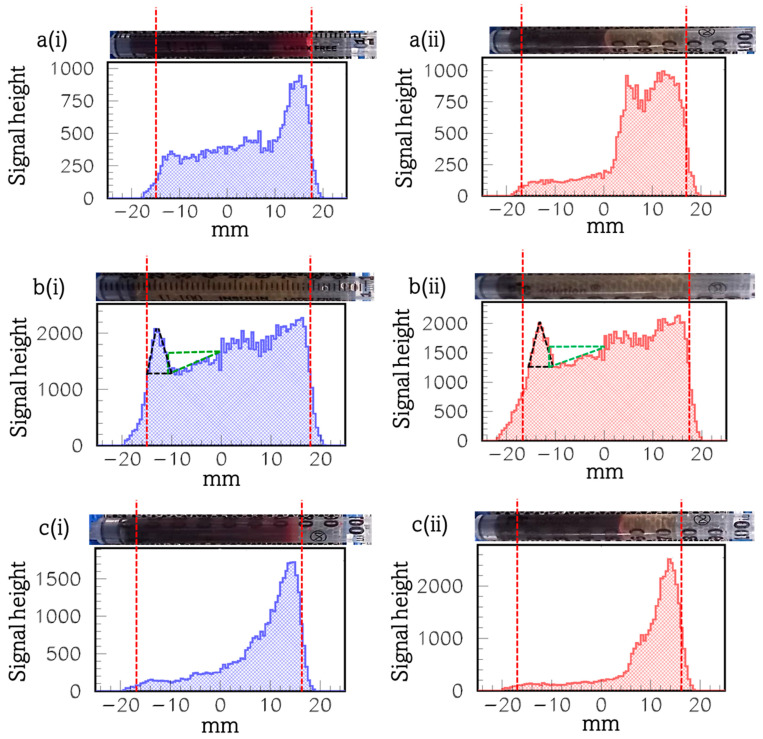
Diagrams of the signal height profile (that is along the axis of the syringe) of the (**a**(**i**),**a**(**ii**)) twice-purified whole blood originally incubated with DMCA (syringe (1)), (**b**(**i**),**b**(**ii**)) PPP incubated with DMCA (syringe (2)), and (**c**(**i**),**c**(**ii**)) intact whole blood incubated with DMCA (syringe (3)), (**a**(**i**),**c**(**i**)) before and (**a**(**ii**),**c**(**ii**)) after centrifugation (donor 3). Above each panel, a photograph of the respective syringe is shown in horizontal position for the sake of presentation. The vertical red dotted lines mark the lower and upper ends of the signal’s distribution and the respective physical bounds of the sample placed inside each syringe. The area of the black triangle corresponds to the signal peak at the bottom of the syringe, while the area of the green triangle is the deficit of the signal evident at the low middle-part of the syringe.

Figure 2 presents the diagrams of the signal height profile (that is along the axis of the syringe) of the Figure 2a(i),a(ii) twice-purified whole blood originally incubated with DMCA (syringe (1)); b(i),b(ii) PPP incubated with DMCA (syringe (2)); c(i),c(ii) intact whole blood incubated with DMCA (syringe (3)); and a(i),c(i) before and a(ii),c(ii) after centrifugation for donor 3 (see the respective hematological data in Table 2, above). The respective photograph of each syringe is placed above the relevant diagram (in horizontal position with its bottom/top on the left/right). 

To begin with, panels a(i),a(ii) demonstrate the twice-purified whole blood originally incubated with DMCA (syringe (1)) a(i) before and a(ii) after its centrifugation at 1200× *g* for 10 min (see Section 2.5 “Gamma-camera imaging of samples”). Comparing the diagrams of panels a(i),a(ii), we observe signal peaks coming from the sample at the top of the syringe. We note during the first imaging (before centrifugation), all cells are inevitably precipitated, at least partially, as they remain under the gravity force, forming a step-like gradient of their concentration along the axis of the syringe (panel a(i)). As a consequence, the respective signal height profile along the axis of the syringe also forms a step-like gradient resulting in a signal peak at the top of the syringe. This indicates that there is higher radioactivity at the top of the syringe and it is an indication of the presence of the DMCA in the supernatant plasma. This result is even more evident in the case of panel a(ii), where the sample after centrifugation is illustrated. In these centrifugation conditions (1200× *g*, 10 min), practically all cells have been precipitated at the bottom of the syringe. The respective signal height profile is low at the same part of the syringe. On the other hand, the signal is relatively much higher at the top of the syringe where plasma accumulates. These results clearly prove the DMCA mainly concentrates in plasma, while it is not delivered to the cellular components of blood.

The panels c(i),c(ii) demonstrate the intact whole blood incubated with DMCA (syringe (3)) sample c(i) before and c(ii) after its centrifugation at 1200× *g* for 10 min. Comparing both diagrams of the panels c(i),c(ii), we observe a qualitatively similar, however, a quantitatively more intense, behaviour that further clarifies the results of the respective panels a(i),a(ii). In both cases, there were intense signal peaks coming from the sample at the top of the syringe, clearly indicating the DMCA has mostly been accumulated in the plasma rather than in the cells, as we previously described.

Compared to the twice-purified whole blood originally incubated with DMCA sample (panels a(i),a(ii)), the intact whole blood incubated with DMCA sample (panels c(i),c(ii)) had higher radioactivity, since it has not been subjected to purification (see Section 2.4 “Preparation of samples”). Accordingly, in panels c(i),c(ii), the respective signal height profile resulted in a high signal peak coming from the plasma area and a much lower signal intensity coming from the cells, compared to the one of panels a(i),a(ii). This result enables us to safely conclude that indeed the DMCA remains in the plasma, even after its incubation for 2 h with the peripheral whole blood and is not attached or internalized in the RBCs.

The panels b(i),b(ii) demonstrate the PPP incubated with DMCA (syringe (2)) sample, b(i) before and b(ii) after its centrifugation at 1200× *g* for 10 min. A simple comparison of both panels indicates similar behaviour. As it is illustrated in both cases, we observe peaks of the signal coming from the plasma at the bottom of the syringe. This result is due to a minor precipitation of the DMCA at the bottom of the syringe. Furthermore, the deficit that is evident at the low middle-part of the syringe (from −10 mm up to 0 mm) in both b(i),b(ii), refers to the DMCA that is being precipitated at the bottom. On a quantitative basis, in panel b(i), the area of the black triangle ((Base × Height)/2~(5 × 700)/2 = 1750) which corresponds to the signal peak at the bottom of the syringe is practically equal to the area of the orthogonal green triangle ((Base × Height)/2~(10 × 400)/2 = 2000) which corresponds to the deficit of the signal evident at the low middle-part of the syringe. The same result stands for panel b(ii), where the area of the black triangle (signal peak) and of the orthogonal green triangle (signal deficit) are 1750 and 1925, respectively. 

**Figure 3:**
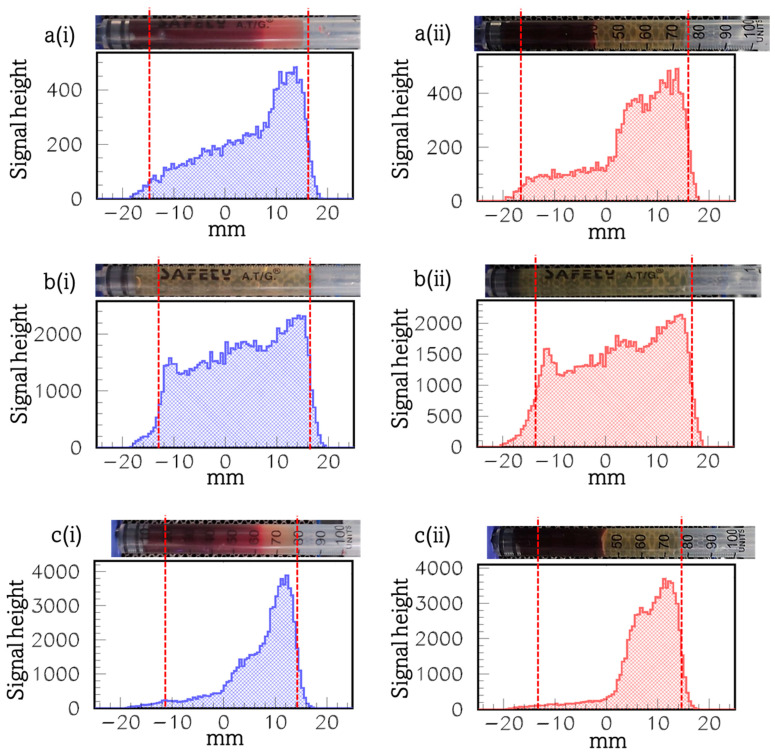
Diagrams of the signal height profile (that is along the axis of the syringe) of the (**a**(**i**),**a**(**ii**)) twice-purified whole blood originally incubated with DMCA (syringe (1)), (**b**(**i**),**b**(**ii**)) PPP incubated with DMCA (syringe (2)), and (**c**(**i**),**c**(**ii**)) intact whole blood incubated with DMCA (syringe (3)), (**a**(**i**),**c**(**i**)) before and (**a**(**ii**),**c**(**ii**)) after centrifugation (donor 2). Above each panel a photograph of the respective syringe is shown in horizontal position for the sake of presentation. The vertical red dotted lines mark the lower and upper ends of the signal’s distribution and the respective physical bounds of the sample placed inside each syringe.

Figure 3 presents the diagrams of the signal height profile (that is along the axis of the syringe) of the Figure 3a(i),a(ii) twice-purified whole blood originally incubated with DMCA (syringe (1)), b(i),b(ii) PPP incubated with DMCA (syringe (2)), and c(i),c(ii) intact whole blood incubated with DMCA (syringe (3)), a(i),c(i) before and a(ii),c(ii) after centrifugation, for donor 2 (see the respective hematological data in Table 2, above). The respective photograph of each syringe is placed above the relevant diagram (in horizontal position with its bottom/top on the left/right).

The explanation to our findings, which were shown in the panels a(i),a(ii),c(i),c(ii) of Figure 3, was similar to the one we previously described for the panels a(i),a(ii),c(i),c(ii) of Figure 2, respectively. Briefly, the intense signal peaks coming from the sample at the top of the syringe, not only in the panels a(i),a(ii), but also in c(i),c(ii) of Figure 3, clearly demonstrated the DMCA has mostly been accumulated in the plasma rather than in the cells.

**Figure 4:**
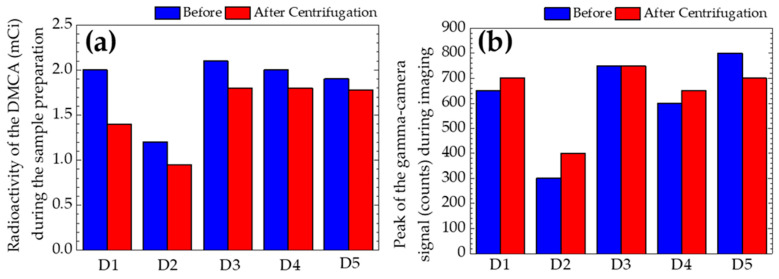
(**a**) The radioactivity (mCi) of the DMCA before and after its centrifugation, which it undergoes during its preparation process (that is referred to the Section 2.4 “Preparation of samples”). (**b**) The peaks of the gamma-signal (counts) coming from the DMCA, which precipitates at the bottom of the syringe before and after centrifugation of the autologous blood plasma incubated with DMCA sample during imaging (that is referred to the Section 2.5 “Gamma-camera imaging of samples”). The five donors are denoted as D1–D5.

The panels Figure 3b(i),b(ii) demonstrate the PPP incubated with DMCA (syringe (2)) sample, b(i) before and b(ii) after its centrifugation at 1200× *g* for 10 min. A simple comparison of both panels indicates similar behaviour. For this specific case, the signal distribution show linear change along the axis of the syringe. Moreover, the signal peaks coming from the plasma at the bottom of the syringe were significantly lower compared to the ones we observed in the respective panels b(i),b(ii) of Figure 2. This result may be attributed to the fact that the radioactivity of the DMCA for this specific donor was found to be 0.95 mCi, before the preparation of the blood and plasma samples. This radioactivity was the smallest during our experiments (see Figure 4). This result is explained as follows: it is well-known ^99m^Tc is obtained from a ^99^Mo/^99m^Tc generator. Specifically, the generator is eluted to provide ^99m^Tc and this can be performed several times a day. Furthermore, the maximum radioactivity of ^99m^Tc is obtained after 24 h. Since the output of the generator declines with time, it needs to be replaced every week. Thus, the low DMCA radioactivity found in donor 2 could be related either to the time of the elution during the day of the conduction of the experiment for this donor or to the fact that the generator had not been replaced before the experiment. According to Figure 4, the higher the radioactivity (mCi) of the DMCA is, the higher are the observed signal gamma-camera peaks (counts) coming from the DMCA, which precipitates at the bottom of the syringe before and after centrifugation. At this point, we should clarify the terms “before” and “after” centrifugation for each of the panels 4(a) and 4(b). In the first case of panel 4(a), we refer to the radioactivity (mCi) of the DMCA before and after its centrifugation, which it undergoes during its preparation process (that is referred to the Section 2.4 “Preparation of samples”). In the second case of panel 4(b), we refer to the peaks of the gamma-signal (counts) coming from the DMCA, which precipitates at the bottom of the syringe, before and after the centrifugation of the PPP incubated with DMCA sample during imaging (that is referred to the Section 2.5 “Gamma-camera imaging of samples”).

Although the sample (n = 5) of donors was small, consistent results were obtained from all donors, as it was indicated by this introductory study. Table 3 presents: (i) the mean value (±SD) for each donor of the radioactivity (mCi) of the DMCA, which is calculated by taking into account the radioactivities before (blue column of Figure 4a) and after DMCA’s centrifugation (red column of Figure 4a) during its preparation, and (ii) the mean value (±SD) for each donor of the gamma-camera signal peaks coming from the DMCA precipitated at the bottom of the syringe. This mean value (±SD) was calculated by taking into account the values before (blue column of Figure 4b) and after (red column of Figure 4b) centrifugation of the PPP incubated with DMCA sample during imaging. 

The mean values of the radioactivity of the DMCA (second column of Table 3) were tested for correlation with the mean values of the peaks of the signal (third column of Table 3). The correlation coefficient between these data was found to be 0.93, showing a positive correlation among them. This result indicates the higher the radioactivity of the DMCA is during its preparation, the higher is the peak of the signal of the DMCA, which precipitates at the bottom of the syringe during imaging.

## 4. Conclusions

In this study, we investigated the distribution of the ^99m^Tc-DPD-Fe_3_O_4_ DMCA in the plasma and cellular components of peripheral human blood. To this end, we employed a gamma-camera apparatus for the direct imaging of twice-purified whole blood originally incubated with DMCA, PPP incubated with DMCA, and intact whole blood incubated with DMCA samples when subjected to specialized centrifugation protocols. The direct comparison of the gamma-camera data obtained at the exact same samples before and after their centrifugation enabled us to survey the distribution of ^99m^Tc-DPD-Fe_3_O_4_. Our results showed the DMCA predominantly concentrated in the plasma with only minor distribution (if any) in the cells. This finding is crucial for the overall evaluation of the biocompatibility of the specific DMCA before planning its use in preclinical practice. Similar studies in other DMCAs would be beneficial as well. 

## Figures and Tables

**Table 1:** Basic information (sex and age) of the donors who participated in the study.

Donors	Sex	Age (Years)
Donor 1	Male	50
Donor 2	Female	60
Donor 3	Female	27
Donor 4	Male	54
Donor 5	Female	32

**Table 2:** Complete blood count examination of five healthy donors. The Table indicates the basic whole blood indices examined along with their physiological range and the respective results obtained for each donor.

Donors	Whole Blood Indices	Physiological Range	Results
Donor 1	Red blood cells (RBCs)	4.50–5.70 M/μL	5.14
	Hemoglobin (HGB)	11–17 g/dL	16.00
	Hematocrit (HCT)	35–55%	44.90
	Mean corpuscular volume (MCV)	76–97 fL	87.40
	Mean corpuscular hemoglobin concentration (MCHC)	26–37 g/dL	35.70
	Mean corpuscular hemoglobin (MCH)	26–36 pg	31.20
	Red cell distribution width (RDW-CV)	11.00–16.00%	13.80
	White blood cells (WBCs)	4.00–11.00 K/μL	6.60
	Platelets (PLTs)	150–400 K/μL	237
	Mean platelet volume (MPV)	6.00–12.00 fL	8.70
	Plateletcrit (PCT)	0.17–2.82%	2.06
Donor 2	Red blood cells (RBCs)	4.50–5.70 M/μL	4.52
	Hemoglobin (HGB)	11–17 g/dL	13.90
	Hematocrit (HCT)	35–55%	42.80
	Mean corpuscular volume (MCV)	76–97 fL	94.60
	Mean corpuscular hemoglobin concentration (MCHC)	26–37 g/dL	32.50
	Mean corpuscular hemoglobin (MCH)	26–36 pg	30.80
	Red cell distribution width (RDW-CV)	11.00–16.00%	12.40
	White blood cells (WBCs)	4.00–11.00 K/μL	6.04
	Platelets (PLTs)	150–400 K/μL	317
	Mean platelet volume (MPV)	6.00–12.00 fL	7.80
	Plateletcrit (PCT)	0.17–2.82%	2.01
Donor 3	Red blood cells (RBCs)	4.50–5.70 M/μL	4.90
	Hemoglobin (HGB)	11–17 g/dL	13.20
	Hematocrit (HCT)	35–55%	41.30
	Mean corpuscular volume (MCV)	76–97 fL	83.80
	Mean corpuscular hemoglobin concentration (MCHC)	26–37 g/dL	32.00
	Mean corpuscular hemoglobin (MCH)	26–36 pg	26.80
	Red cell distribution width (RDW-CV)	11.00–16.00%	13.10
	White blood cells (WBCs)	4.00–11.00 K/μL	5.40
	Platelets (PLTs)	150–400 K/μL	229
	Mean platelet volume (MPV)	6.00–12.00 fL	8.60
	Plateletcrit (PCT)	0.17–2.82%	0.42
Donor 4	Red blood cells (RBCs)	4.50–5.70 M/μL	4.59
	Hemoglobin (HGB)	11–17 g/dL	14.30
	Hematocrit (HCT)	35–55%	43.00
	Mean corpuscular volume (MCV)	76–97 fL	93.00
	Mean corpuscular hemoglobin concentration (MCHC)	26–37 g/dL	33.40
	Mean corpuscular hemoglobin (MCH)	26–36 pg	31.10
	Red cell distribution width (RDW-CV)	11.00–16.00%	13.90
	White blood cells (WBCs)	4.00–11.00 K/μL	7.73
	Platelets (PLTs)	150–400 K/μL	187
	Mean platelet volume (MPV)	6.00–12.00 fL	13.70
	Plateletcrit (PCT)	0.17–2.82%	0.26
Donor 5	Red blood cells (RBCs)	4.50–5.70 M/μL	4.78
	Hemoglobin (HGB)	11–17 g/dL	12.8
	Hematocrit (HCT)	35–55%	39.9
	Mean corpuscular volume (MCV)	76–97 fL	83.50
	Mean corpuscular hemoglobin concentration (MCHC)	26–37 g/dL	32.08
	Mean corpuscular hemoglobin (MCH)	26–36 pg	26.78
	Red cell distribution width (RDW-CV)	11.00–16.00%	14.20
	White blood cells (WBCs)	4.00–11.00 K/μL	8.20
	Platelets (PLTs)	150–400 K/μL	269
	Mean platelet volume (MPV)	6.00–12.00 fL	7.50
	Plateletcrit (PCT)	0.17–2.82%	0.21

**Table 3:** Mean value ± standard deviation (MV ± SD) for each donor of the radioactivity (mCi) of the DMCA, which was calculated by taking into account the radioactivities before (blue column of Figure 4a) and after DMCA’s centrifugation (red column of Figure 4a) during its preparation. Mean value ± standard deviation (MV ± SD) for each donor of the gamma-camera signal peaks coming from the DMCA found at the bottom of the syringe. It was calculated by taking into account the values before (blue column of Figure 4b) and after (red column of Figure 4b) centrifugation of the PPP incubated with DMCA sample during imaging. Both columns of MV were analyzed statistically with correlation test.

Donors	Radioactivity of the DMCA (mCi) (MV ± SD)	Peak of the Signal (MV ± SD)
Donor 1	1.70 ± 0.42	675 ± 35
Donor 2	1.08 ± 0.18	350 ± 71
Donor 3	1.95 ± 0.21	750 ± 0
Donor 4	1.90 ± 0.14	625 ± 35
Donor 5	1.84 ± 0.08	750 ± 71

## Data Availability

The data presented in this study are available upon request from the corresponding authors M.-A.K. and D.S.
